# Acute total hip replacement of acetabular fractures with cementless modular revision cups in patients older than 55 years: a retrospective cohort study

**DOI:** 10.1007/s00068-025-03045-9

**Published:** 2025-12-18

**Authors:** Mohammed Rashed, Frank Hildebrand, Ulf Hofmann, Klemens Horst, Benedikt Hürtgen, Eftychios Bolierakis, Till Berk, Hatem Alabdulrahman

**Affiliations:** 1 Department of Orthopaedic Surgery, University Hospital Aachen, Pauwelsstraße 30, 52074 Aachen, Germany; 2 Department of Orthopaedic Surgery, Zagazig General Hospital, Zagazig, Egypt

**Keywords:** Acute total hip replacement, Acetabular fractures, Cementless modular prosthesis, Osteosynthesis, Combined hip procedure, Decision-making

## Abstract

**Purpose:**

To compare early postoperative results for the management of acetabular fractures using open reduction and internal fixation versus acute total hip replacement with a cementless modular cup in patients older than 55 years.

**Methods:**

This was a retrospective cohort study conducted at a Level I trauma center between April 2019 and March 2024, including 36 consecutive patients aged ≥ 55 years with displaced acetabular fractures treated by a single senior pelvic surgeon using open reduction and internal fixation (*n* = 20), stand-alone total hip replacement (*n* = 9), or a planned two-stage combined hip procedure (*n* = 7). The primary outcome included Harris Hip Score at 6 months, Secondary outcomes included postoperative complications, unplanned reoperation, and radiographic implant assessment.

**Results:**

At six months, both replacement groups showed significantly better functional outcomes (mean Harris hip scores of 83 and 82) compared to the fixation-only group (mean score of 57; *p* < 0.001). All reoperations (4/20) occurred in the fixation group, while no reoperation was required in the replacement groups. Radiographic evaluation confirmed stable implant fixation in all arthroplasty cases, without signs of loosening or dislocation.

**Conclusion:**

According to our treatment algorithm, acute total hip replacement using a modular revision cup with an iliac peg, with or without plate osteosynthesis, offers the advantage of early full weight-bearing and promising functional outcomes in the management of complex acetabular fractures. These results support the integration of acute total hip replacement into structured decision-making protocols especially for elderly patients.

## Introduction and background

The incidence of acetabular fractures (AFs) in the elderly population has more than doubled over the past three decades [1]. At present, around half of all patients with AF are aged over 60 years [2]. The prevalence of these injuries is also projected to increase by 140% within the next five-years due to current demographic changes [3].

In elderly patients, even low-energy trauma can lead to complex patterns of AF [4]. Anterior column displacement, quadrilateral plate (QLP) involvement, and articular impaction characterise a substantial proportion of AFs in this population [5]. In such cases, preserving the native hip joint becomes increasingly challenging, particularly in the presence of osteoporosis, severe comminution, or pre-existing hip osteoarthritis [4].

As a result, open reduction and internal fixation (ORIF) of AF has often been associated with relatively poor outcomes [6]. Approximately 23% of patients over 55 ultimately require conversion to secondary total hip replacement (THR), which often yields inferior results compared to acute THR [7].

These limitations of ORIF in elderly patients with AF highlight the necessity for updated treatment strategies with a clear decision-making algorithm [8]. Primary THR, either alone or combined with plate osteosynthesis, has gained ground as a promising alternative to ORIF in elderly patients [9]. This approach may minimize the need for reoperation and enable early mobilization, which is essential for maintaining functional independence and preventing complications [6, 10].

The aim of this study was to evaluate the clinical performance of acute THR using a cementless modular revision cup compared with ORIF in elderly patients with AFs. We hypothesized that acute THR would achieve superior functional recovery at six months, as measured by the Harris Hip Score (HHS), while maintaining a comparable safety profile to ORIF.

## Methods

### Ethical considerations

This study was approved by the local ethics committee (Ethics Committee at the RWTH Aachen Faculty of Medicine, Protocol No. EK 25–247). The study was conducted in accordance with the ethical principles of the seventh revision of the Declaration of Helsinki and the Good Clinical Practice Guidelines. Due to the purely retrospective nature of the study and the subsequent use of anonymized data, the requirement for written informed consent was waived by the ethics committee in compliance with institutional and national data protection regulations.

### Study design

This retrospective cohort study was conducted at a Level I trauma centre over a five-year period, from April 2019 to March 2024. Data were extracted from electronic medical records and radiological archives.

### Inclusion and exclusion criteria

Patients were identified using an administrative database search based on relevant ICD-10 codes related to AF. Patients aged 55 years or older with AF who underwent operative treatment were considered eligible. Only cases managed by the same senior surgeon and having a minimum follow-up period of six months were included.

Patients were excluded if they were under 55 years of age, treated by other surgeons, had periprosthetic or pathological fractures, suffered concomitant injuries affecting weight-bearing capacity, had severe neurological disorders influencing postoperative mobility, or had a follow-up period of less than six months.

### Study groups

Patients were divided into three groups according to the surgical treatment received:

1.  stand-alone acute total hip replacement **(SA-THR)**, defined as primary arthroplasty performed during the index admission for a traumatic AFs without prior fixation.
2.  combined hip procedure **(CHP)**, a planned two-stage strategy in which the first operation involved internal fixation of the acetabular columns to restore stability, followed by a second operation for cementless total hip replacement using a modular revision cup. Both stages were performed during the same hospital admission, with the interval between procedures determined by the patient’s clinical condition and soft-tissue recovery.
3. open reduction and internal fixation **(ORIF)**.

Operational decisions for elderly AF are made at the study location according to a standard operative procedure (SOP) algorithm, considering age, bone quality, joint condition, and comorbidities. Additionally, this approach combines established treatment principles and published indications for acute THR in AF with our own recommendations based on fracture morphology to ensure individualized evidence-based care.

**Fig. 1:**
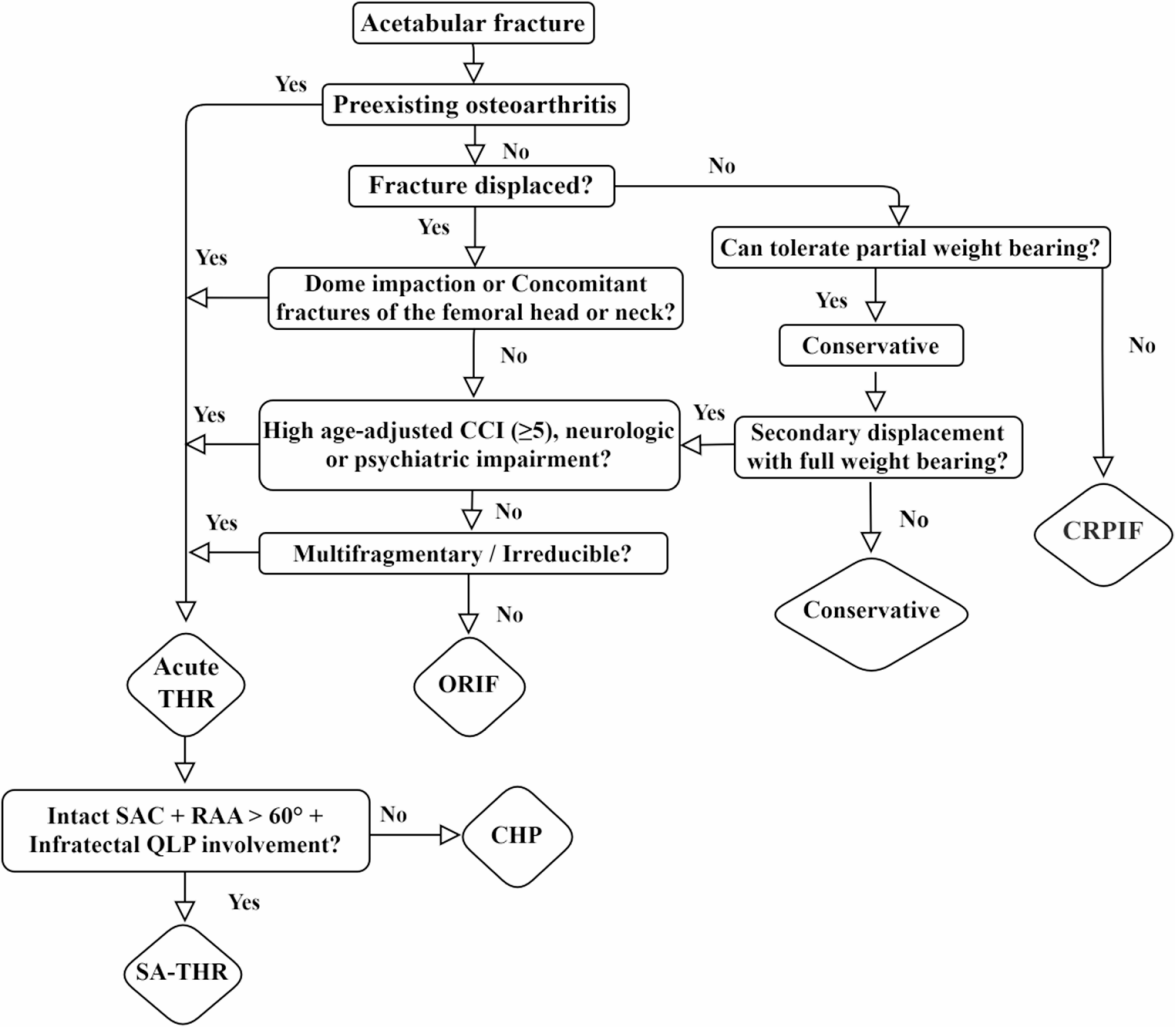
Decision-making algorithm for the management of acetabular fractures in the elderly. CRPIF = Closed Reduction and Percutaneous Internal Fixation

Surgical strategy was determined by the main senior pelvic surgeon (last author), to guide the choice between ORIF, SA-THR, and CHP (Fig. 1). ORIF was chosen for patients with anatomically reducible fractures in the absence of significant pre-existing hip osteoarthritis.

In contrast, acute THR was preferred in fractures with complex patterns, as seen in anterior column–posterior hemitransverse (AC-PHT) fractures with dome impaction, or multifragmentary posterior wall fractures [11]. This strategy was especially considered in patients with osteoporosis or a high age-adjusted Charlson Comorbidity Index (CCI ≥ 5) [12]. In such cases, even achieving anatomical reduction was unlikely to ensure a satisfactory outcome, given the need for prolonged weight-bearing restrictions [7].

SA-THR was performed when the supracetabular corridor (SAC) was intact, the posterior roof arc angle (RAA) exceeded 60° and in fractures with infratectal QLP involvement (Fig. 1). CHP was indicated for trans- and juxta‑tectal fractures or cases where the posterior RAA was less than 60° [13]. CHP was implemented in two stages (fix and replace) to minimize the surgical burden on elderly patients while ensuring optimal fracture stabilization [6].

### Documented data

Demographic and clinical data were systematically recorded, including age, gender, and body mass index (BMI). Clinical characteristics included the CCI and osteoporosis, recorded based on the documented patient medical history. Further retrospective evaluation included injury severity score (ISS), length of hospital stay (LOS), and time from admission to surgery (TTS).

Operative records were screened to extract surgical duration and fluoroscopy time. Intraoperative blood loss was estimated using the Gross formula [14].

Pre- and postoperative imaging was extracted from the hospital’s radiological system (Philips IntelliSpace PACS). Standard X-rays in anteroposterior (AP), inlet, outlet, and Judet views, as well as CT scans, were evaluated. Fractures were classified according to the Judet-Letournel classification system. Fracture morphology was assessed in terms of comminution, QLP involvement, presence of gull sign, dome impaction, head migration/dislocation, and RAA according to Matta [15].

### Surgical technique

In the SA-THR group, a modular revision cup (MRS-Titan^®^ maximum, Peter Brehm GmbH) was used and secured with a 70 mm iliac peg (10 mm diameter) at a 70° angle. Three flat-head spongiosa screws of 6.5 mm were placed in the strap areas, and one screw was inserted in the dome. The surgical approach used in THR was a modified Hardinge approach performed with the patient in the lateral decubitus position. In the THR groups, Actinia^®^ femoral stem (Implantcast GmbH) was used in all cases.

In the CHP group, a PRO Quadrilateral Surface Plate (Stryker GmbH & Co. KG) was used before THR to provide medial wall support. The plate has 16 screw holes, with the medial row filled with three to four 3.5 mm screws, while the lateral row was fitted with three 3.5 mm screws. Fixation was performed through the anterior intrapelvic approach (AIP) with the patient in the supine position.

**Fig. 2:**
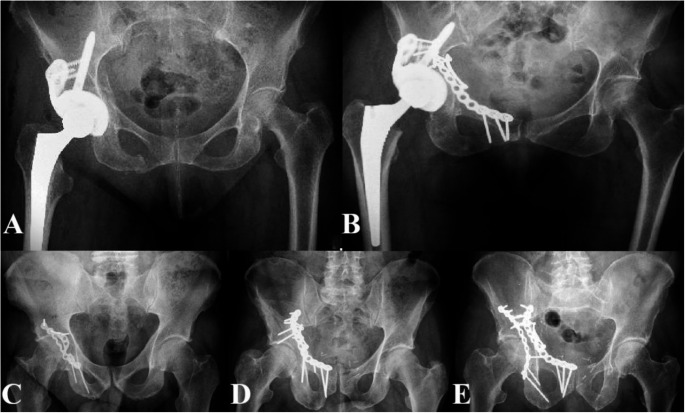
X-ray images extracted from the hospital’s radiology system (Philips IntelliSpace PACS) representing the implants used in each group: (**A**) SA-THR group – modular revision cup; (**B**) CHP group – PRO Quadrilateral Surface Plate prior to THR; (**C**) ORIF group – posterior wall fixation using a pre-contoured 3.5 mm plate; (**D**) ORIF group – anterior column fixation using a PRO Quadrilateral Surface Plate; (**E**) ORIF group – combined anterior column and posterior column fixation using a PRO Quadrilateral Surface Plate and a low-profile posterior plate

In the ORIF group, patients received either anterior fixation through the AIP approach, posterior fixation via the Kocher-Langenbeck approach with the patient in the lateral decubitus position, or both column fixation depending on the fracture morphology. The anterior fixation was performed using PRO Quadrilateral Surface Plate. Posterior column fractures were stabilized with a low-profile 3.5 mm pelvic recon plate (Synthes), while posterior wall fixation was performed using a pre-contoured 3.5 mm plate (I.T.S. GmbH) (Fig. 2).

### Postoperative analysis and treatment

Postoperative X-rays were analyzed to assess acetabular cup orientation focusing on inclination angle, cup anteversion, and centre of rotation (CoR) relative to the normal side. Limb-length discrepancy (LLD) was assessed using postoperative pelvic radiographs [16]. Postoperative radiological outcomes in the ORIF group were evaluated, including maximal residual displacement (MRD) according to Matta’s criteria [17].

Each group underwent postoperative rehabilitation according to its respective SOP. THR patients were allowed to bear full weight as tolerated from the first postoperative day, while walking aids were generally recommended during the first six weeks [18]. ORIF patients were advised to follow a partial weight-bearing protocol (up to 15 kg) using walking aids for the first 8 weeks postoperatively, followed by progressive weight-bearing as tolerated [19].

### Outcome

All postoperative outcomes, including complications and functional scores, were analyzed within a standardized 6-month postoperative window to ensure uniform observation time and avoid bias from unequal follow-up durations.

The primary outcome was functional recovery, assessed by the Harris Hip Score (HHS). The six-month score served as the primary endpoint, while additional measurements at one and six weeks were recorded to evaluate recovery over time. Secondary outcomes included early postoperative complications, unplanned reoperations within six months, and radiographic implant assessment.

Postoperative complications were identified through a systematic review of clinical and radiological records, including inpatient notes, discharge summaries, and follow-up documentation. Each event was independently verified and recorded once per patient to avoid duplication. Because of the small sample size and the presence of zero-event categories, the two arthroplasty cohorts, SA-THR and the CHP were pooled into a single THR group for complication analysis to ensure statistical validity and meaningful interpretation.

Complications were categorized as follows: pulmonary (pneumonia or respiratory insufficiency), urinary tract infection (UTI), cognitive (postoperative delirium), wound/implant-related (surgical-site infection or intra-articular screw), and neurovascular (sciatic nerve or external iliac vein injury). Deep vein thrombosis (DVT) and pulmonary embolism were included only when clinically documented, as no routine screening was performed. Unplanned reoperation was defined as any secondary surgical intervention related to the index acetabular procedure within six months, excluding the planned second stage of the CHP protocol.

Postoperative serial X-rays were used to monitor osteoarthritis progression, assessed using the Tönnis classification [20]. Heterotopic ossification was classified according to Brooker et al. [21]. Implant stability and component positioning were evaluated in the arthroplasty groups.

### Statistical analysis

Statistical analysis was performed using SPSS 28 (IBM Corp., Armonk, NY, USA). Normality was assessed using the Shapiro–Wilk test. For comparisons among the three groups, the Kruskal–Wallis test was applied for continuous variables, and Fisher’s Exact Test for categorical variables. Mann–Whitney U tests were used for non-parametric pairwise comparisons, while unpaired t-tests were used for normally distributed continuous data.

Effect sizes were calculated to assess the strength of association between treatment group and the primary outcome (Harris Hip Score, HHS). For each Kruskal–Wallis test across the three groups (ORIF, SA-THR, CHP), the epsilon-squared statistic (ε²) was computed using the formula ε² = (H − k + 1)/(N − k), where H is the Kruskal–Wallis test statistic, k is the number of groups, and N is the total sample size. For pairwise Mann–Whitney U comparisons, the rank-biserial correlation (r) was derived from the Z value using the formula r = |Z|/√N. Effect size thresholds were interpreted as small (≈ 0.01–0.13), medium (≈ 0.13–0.26), and large (≥ 0.26) [22].

## Results

### Study population

**Fig. 3:**
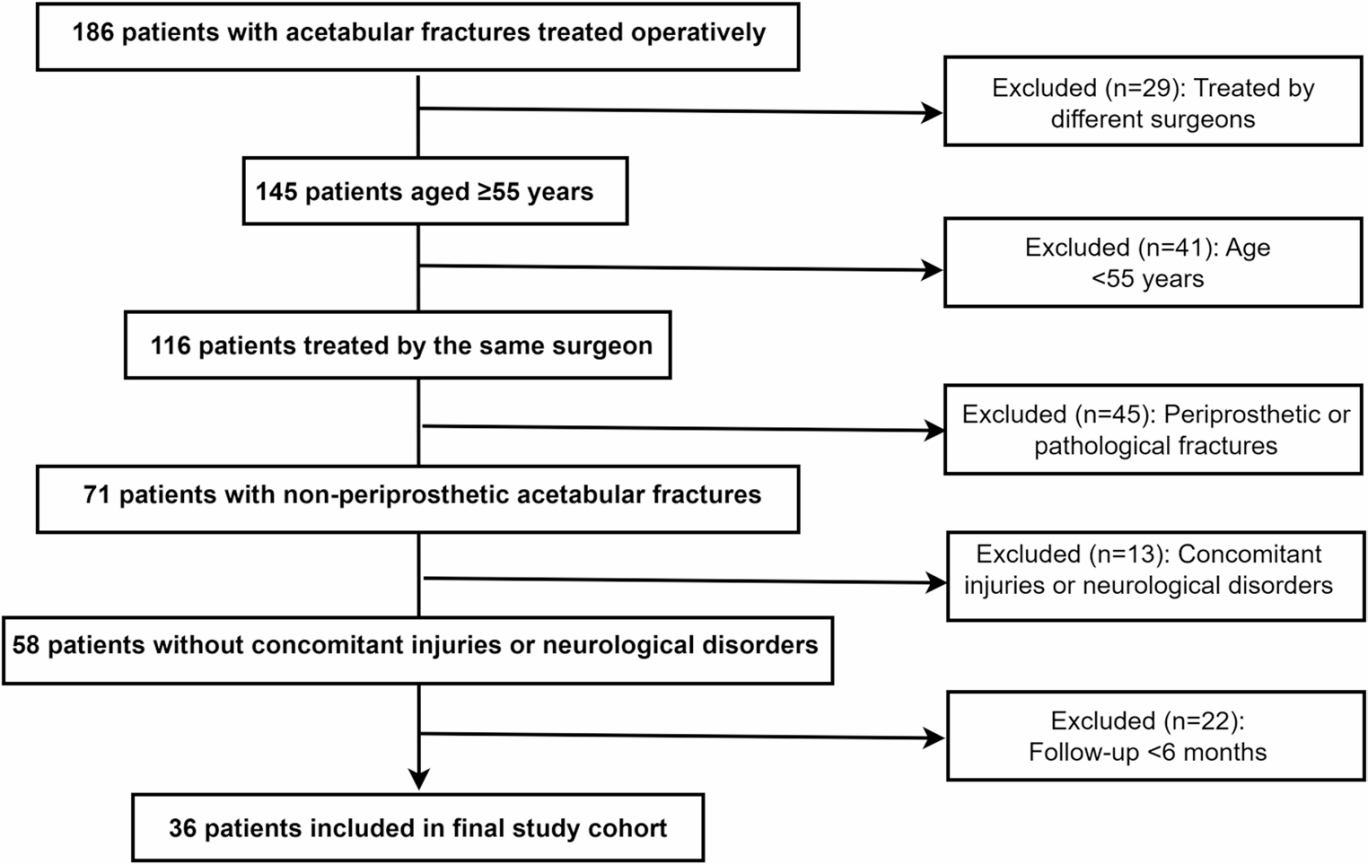
Patient selection process leading to the final study cohort of 36 patients

A total of 186 patients with AFs were screened. After applying inclusion and exclusion criteria, 36 patients were eligible for analysis (Fig. 3).

Among the included patients, 7 were treated with CHP, 9 underwent SA-THR, and 20 received ORIF. Within the ORIF group, 14 patients had anterior fixation, 3 had posterior fixation, and 3 underwent both column fixation.

### Baseline characteristics

The mean age showed a numerical trend toward lower values in the ORIF group, although the difference was not statistically significant (*p* = 0.162). Both the SA-THR and CHP groups demonstrated a significantly higher CCI compared with ORIF (*p* = 0.033). No relevant differences were observed among the groups regarding BMI, osteoporosis, ISS, LOS, or TTS. In contrast, the ORIF group had a markedly longer follow-up period (*p* < 0.001) (Table 1).

Preoperative imaging analysis revealed variability in fracture morphology between the THR groups and the ORIF group. The anterior column–posterior hemitransverse (AC-PHT) configuration represented the predominant fracture type in both the ORIF and SA-THR groups (12/20 ORIF, 5/9 SA-THR). Other fracture patterns included both-column (BC) fractures (3/20 ORIF), posterior wall (PW) fractures (3/20 ORIF, 2/9 SA-THR), and single transverse (Trans) and T-type fractures (1/20 each ORIF). In the CHP group, fracture morphology differed, consisting of anterior wall–posterior hemitransverse (AW-PHT, 3/7), transverse–posterior wall (Trans-PW, 2/7), and anterior column–posterior hemitransverse (AC-PHT, 2/7) patterns. Dome impaction and the Gull sign were observed significantly more often in the CHP group, indicating the highest level of fracture complexity (Table 1).

**Table 1:** Baseline characteristics and fracture morphology of patients treated with ORIF, SA-THR, or CHP

Variable	SA-THR (*n* = 9)	CHP (*n* = 7)	ORIF (*n* = 20)	*P*-valueᵃ
Age (years)ᵇ	78 ± 12	75 ± 10	70 ± 11	0.162
Gender				0.161
Male (%)	5 (55.6%)	3 (42.9%)	15 (75.0%)	
Female (%)	4 (44.4%)	4 (57.1%)	5 (25.0%)	
BMI (kg/m²)ᵇ	25 ± 7	24 ± 4	28 ± 5	0.113
Osteoporosis (%)	2 (22.2%)	2 (28.6%)	1 (5.0%)	0.377
CCIᶜ	5 (4–6)	4 (2–4)	3 (1–4)	**0.033**
ISSᶜ	4 (4–9)	4 (4–9)	4 (4–9)	0.974
LOS (days)ᶜ	13 (9–22)	20 (12–31)	16 (11–26)	0.422
TTS (days)ᶜ	5 (4–7)	13 (2–23)	6 (5–9)	0.628
Follow-up period (weeks)ᶜ	24 (24–35)	24 (24–26)	43 (29–77)	**< 0.001**
Fracture morphology				
QLP fracture (%)	7 (77.8%)	7 (100%)	15 (75.0%)	0.443
Multifragmentary fracture (%)	4 (44.4%)	2 (28.6%)	2 (10.0%)	0.067
Gull sign (%)	4 (44.4%)	7 (100%)	9 (45.0%)	**0.027**
Dome impaction (%)	5 (55.6%)	7 (100%)	9 (45.0%)	**0.034**

Data are shown as mean ± SD (ᵇ), median (IQR) (ᶜ),or number (%). *P*-values (ᵃ) were obtained using the Kruskal–Wallis test for continuous and the Fisher’s exact test for categorical variables.Post-hoc Dunn–Bonferroni correction was applied when appropriate.Bold values indicate *p* < 0.05

### Surgical data

Osteosynthesis as the first step of the CHP procedure (CHP-Fix) showed a significantly higher intraoperative blood loss compared to the ORIF group (*p* = 0.159), while operative time tended to be longer in the ORIF group without reaching statistical significance(*p* = 0.031). Fluoroscopy duration, transfusion rate, and ICU admission were comparable between groups (Table 2).

**Table 2:** Operative characteristics of the ORIF group versus the fixation stage of the CHP

Variable	CHP-Fix (*n* = 7)	ORIF (*n* = 20)	*P*-value (ᵃ)
Length of Surgery (min) (ᶜ)	97 (96–155)	140 (117–179)	0.159
Fluoroscopy Time (sec) (ᶜ)	99 (80–135)	91 (65–172)	0.507
Blood Loss (ml) (ᶜ)	1198 (949–1760)	775 (499–997)	**0.031**
Blood Transfusion (%)	2/7 (29%)	4/20 (20%)	0.633
ICU Admission (%)	2/7 (29%)	7/20 (35%)	1.000

Data are shown as median (IQR) (ᶜ) or frequencies (%). *P*-values (ᵃ) were calculated using the Mann–Whitney U test for continuous variables and Fisher’s Exact Test for categorical variables

Bold values indicate statistically significant results (*p* < 0.05)

he CHP-Rep procedures showed a tendency toward longer operative duration, but without statistical significance (median 191 vs. 171 min, *P* = 0.055). No meaningful differences were observed regarding fluoroscopy use, intraoperative blood loss, or postoperative course, including transfusion and ICU admission rates. (Table 3).

**Table 3:** Operative characteristics of the isolated THR group (SA-THR) versus the replacement stage (CHP-Rep) of the combined CHP procedure

Variable	CHP-Rep (*n* = 7)	SA-THR (*n* = 9)	*P*-value (ᵃ)
Length of Surgery (min) (ᶜ)	191 (185–199)	171 (166–192)	0.055
Fluoroscopy Time (sec) (ᶜ)	120 (77–191)	129 (101–172)	0.837
Blood Loss (ml) (ᶜ)	877 (768–1318)	797 (421–1167)	0.252
Blood Transfusion (%)	1/7 (14%)	2/9 (22%)	1.000
ICU Admission (%)	2/7 (29%)	4/9 (44%)	0.633

Data are shown as median (IQR) (ᶜ) or frequencies (%). *P*-values (ᵃ) were calculated using the Mann–Whitney U test for continuous variables and Fisher’s Exact Test for categorical variables. A *P*-value < 0.05 was considered statistically significant

### Complications

Pulmonary events, UTIs, and postoperative delirium occurred at comparable rates between the THR and ORIF groups. No cases of DVT or pulmonary embolism were documented in the clinical reports. Wound- or implant-related complications and neurovascular injuries were observed more frequently after ORIF. Unplanned reoperations occurred only after ORIF, indicating a trend toward a lower revision risk following THR, although this difference did not reach statistical significance (*p* = 0.113) (Table 4). No mortality occurred in any patient within the six-month follow-up period.

**Table 4:** Postoperative complications — THR (pooled SA-THR and CHP) vs. ORIF group

Complication composite	THR (*n* = 16)	ORIF (*n* = 20)	*P*-valueᵃ
Pulmonary (pneumonia or respiratory insufficiency)	4 (25.0%)	2 (10.0%)	0.374
UTI	0 (0.0%)	2 (10.0%)	0.492
Cognitive (postoperative delirium)	2 (12.5%)	2 (10.0%)	1.000
Wound/implant-related (surgical-site infection or intra-articular screw)	1 (6.3%)	3 (15.0%)	0.613
Neurovascular (sciatic nerve or external iliac vein injury)	0 (0.0%)	3 (15.0%)	0.238
Unplanned reoperation (per patient)	0 (0.0%)	4 (20.0%)	0.113

Data are shown as frequencies (%). The SA-THR and CHP groups were pooled into a single THR group for complication analysis. *P*-values (ᵃ) were calculated using Fisher’s Exact Test. A *P*-value < 0.05 was considered statistically significant

### Harris hip score

SA-THR and CHP groups showed significantly higher HHS throughout the 6-month follow-up (Table 5). Patients in arthroplasty groups also showed lower pain levels, improved gait, and greater functional recovery throughout the follow-up period. Functional activities, such as stair climbing, sitting, and using socks/shoes, were significantly better at all follow-up points. Notably, no significant functional gap was observed between the SA-THR and CHP groups.

**Table 5:** Comparison of HHS scores among the groups at different postoperative time points

Variable	SA-THR (*n* = 9)	CHP (*n* = 7)	ORIF (*n* = 20)	*p*-valueᵃ	Effect size (ε²)ᵇ
HHS 1 week	62 (62–63)	62 (62–64)	47.5 (37–58)	**< 0.001**	0.64
HHS 6 weeks	72 (69–75)	69 (66–71)	49 (39–63)	**< 0.001**	0.60
HHS 6 months	83 (77–84)	82 (76–85)	57 (37–71)	**< 0.001**	0.37

Data are shown as median (IQR) (ᶜ). *P*-values (ᵃ) were obtained using the Kruskal–Wallis test for continuous variables. Post-hoc Dunn–Bonferroni correction was applied when appropriate. Effect size (ᵇ) expressed as epsilon-squared (ε² = H/[N – 1]); interpreted as small ≈ 0.01–0.13, medium ≈ 0.13–0.26, large ≥ 0.26. A *P*-value < 0.05 was considered statistically significant. Bold values indicate *p* < 0.05

### Post-operative radiological outcomes

Postoperative radiological evaluation showed that while the majority of ORIF patients achieved anatomical reduction based on Matta criteria (MRD), a subset experienced postoperative osteoarthritis progression. Approximately 7 out of 20 patients showed signs of moderate to severe osteoarthritis, with three patients who eventually required secondary THR due to persistent pain or coxarthrosis (Table 6).

**Table 6:** Radiological outcomes in the ORIF group

Item	ORIF Group (*n* = 20)
MRD	
Poor	1 (5%)
Imperfect	4 (20%)
Anatomical	15 (75%)
Mean MRD (mm)ᵇ	0.6 mm
OA Grading (Tönnis Classification)	
Grade 0: Normal	8 (40%)
Grade 1: Minor	5 (25%)
Grade 2: Moderate	3 (15%)
Grade 3: Coxarthrosis	4 (20%)
Heterotopic Ossification	
0	16 (80%)
2	1 (5%)
3	3 (15%)
Secondary THR	3 (15%)

Data are shown as mean ± SD (ᵇ) or frequencies (%). OA was graded according to the Tönnis classification, and heterotopic ossification according to the Brooker classification

Radiographic assessment demonstrated acceptable implant positioning in both SA-THR and CHP groups, with proper cup inclination, combined anteversion, and iliac peg alignment, ensuring effective load distribution. The LLD remained within an acceptable range, and CoR remained well-aligned in both horizontal and vertical planes. At six months, all patients in both groups had stable implants with no loosening, malalignment, or dislocation (Table 7). There were no notable differences between the SA-THR and CHP groups across the evaluated parameters. Combined anteversion was slightly lower in the CHP group, yet still within an acceptable range.

**Table 7:** Postoperative image Analysis – Comparison between SA-THR and CHP

Parameter	SA-THR (n = 9)	CHP (n = 7)	*P*-valueᵃ
Inclination angle (°)ᵇ	40.22 ± 3.70	41.14 ± 2.73	0.580
Iliac peg frontal plane (°)ᵇ	69.8 ± 3.6	68.71 ± 2.98	0.478
Combined anteversion (°)ᵇ	24.2 ± 3.0	21.00 ± 3.21	0.038*
CoR – horizontal (mm)ᵇ	2.3 ± 6.3	3.14 ± 5.67	0.757
CoR – vertical (mm)ᵇ	0.5 ± 6.8	2.00 ± 6.38	0.631
LLD (mm)ᶜ	7.0 (6.0–8.0)	2.0 (0.0–8.5)	0.669
Implant stability (6 months) (%)	9 (100%)	7 (100%)	—

Data are shown as mean ± SD (ᵇ), median (IQR) (ᶜ), or frequencies (%). *P*-values (ᵃ) were calculated using the t-test or Mann–Whitney U test, as appropriate. A *P*-value < 0.05 was considered statistically significant (*)

## Discussion

The increased incidence of elderly AFs represents a substantial challenge in orthopaedic trauma care [2]. Continuous advancements in surgical strategies are mandatory to optimize functional recovery while simultaneously minimizing associated complications [9].

The present study compared the outcomes of ORIF as a traditional management procedure with those of acute THR using a cementless modular revision cup and identified the following major insights:

1. Surgical Risk Assessment: Despite longer surgical duration, as compared to ORIF, acute THR did not pose a higher surgical risk in terms of blood loss, ICU admissions, or postoperative complications.
2. Fracture Morphology & Outcomes: Acute THR demonstrated acceptable outcomes in displaced AFs, especially those associated with articular impaction, supporting its role as a viable primary option in selected cases.
3. Early Weight-Bearing & Functional Benefits: Acute THR facilitates early full weight-bearing and achieves superior functional outcomes compared to ORIF.
4. Modular Cup Stability: A modular cup with an iliac peg may offer sufficient primary stability without additional plate osteosynthesis in fracture patterns that fulfil the predefined criteria described in our treatment algorithm.

Assessing the surgical burden is crucial when considering operative strategies in elderly patients with AFs [3]. The extended surgical duration for acute THR is well-documented in the literature [23]. Adjustment of the acetabular fragment, meticulous reaming, femoral preparation, and optimized implant positioning are additional steps that may require considerable time compared to ORIF [24].

Few studies have reported the use of acute THR without internal fixation for the treatment of acetabular fractures in elly patientsder [25, 26]. McMahon et al. reported a mean operative time of 93 min using a posterior approach with a coned hemipelvis construct in 21 patients, noting that 3 cases required blood transfusion, although total blood loss was not specified [26]. Enocson and Blomfeldt performed standalone THR using Burch‑Schneider reinforcement ring through a modified Hardinge approach in 13 patients, reporting a mean operative time of 149 min and a mean blood loss of 665 mL [25].

In our cohort, standalone THR was also performed using the modified Hardinge approach, with a longer mean operative time and higher intraoperative blood loss compared to the aforementioned studies. This could at least partly be explained by the precise adjustment required for iliac peg placement during modular cup positioning into the SAC.

Over the past decade, many studies addressing acute THR for acetabular fractures in elderly patients have adopted a CHP strategy [23]. Multiple studies have implemented the combined hip procedure using a single surgical approach, most commonly the posterior (Kocher–Langenbeck) approach [6, 25, 27–30]. Among these, the mean operative time ranged from 122.7 min to 264 min [25, 29]. The mean or median intraoperative blood loss ranged from 533 mL to 1100 mL, depending on fracture complexity and implant strategy [27, 28].

A number of studies have utilized a dual surgical approach to perform the combined hip procedure in a single operative session, most commonly combining the anterior intrapelvic (Stoppa or ilioinguinal) with the posterior Kocher–Langenbeck approach or a direct anterior approach [29, 31, 32]. Across these studies, the mean intraoperative blood loss reached up to 2120 mL, and the mean operative time up to 300 min when the dual approach was performed in a single operative session [29, 32]. In this context, staging may help reduce per-session surgical stress, improving hemodynamic control and postoperative recovery, particularly beneficial in elderly or comorbid patients.

To our knowledge, no previous study has yet described a planned two-stage acute THR protocol. Given that our approach involved two separate operations, comparison with single-session procedures in the literature must be interpreted with caution. Each surgical stage in our series was analysed independently to allow stage-specific comparisons. The fixation stage involved anterior column reconstruction via an intrapelvic approach. While the operative time was slightly shorter in the fixation stage, it was associated with greater blood loss compared to the ORIF group. For the replacement stage, blood loss and duration of surgery exceeded the values recorded for the standalone THR group.

The longer surgical time for this second stage most likely reflects the technical challenge of navigating retained hardware during cup placement. The increased blood loss for both stages might be associated with a more complex fracture pattern of CHP-cases. The fracture complexity in the acetabulum has been shown to clearly correlate with increased intraoperative blood loss [33, 34].

Consistent with our algorithm, the CHP strategy in this study was reserved for more complex fracture patterns with extensive involvement of both anterior and posterior acetabular structures. Therefore, the increased operative time and blood loss observed in the CHP group seem to be primarily driven by the underlying fracture severity rather than the procedural design alone. Although the higher blood loss observed in the CHP group did not result in significant differences for blood transfusion rates or ICU admission rates among the groups, they have to be carefully weighed against its procedural advantages, particularly in elderly or comorbid patients.

In our study, age and Charlson Comorbidity Index (CCI) were significantly higher in the SA-THR and CHP groups compared to ORIF. Nevertheless, postoperative complication rates remained similar across all treatment strategies, and no mortality was observed at six months. Previous studies report differing mortality rates after acetabular fracture surgery in the elderly. A systematic review reported 1-year mortality of 22.6% after ORIF and 8.8% after THR [35]. A meta-analysis found overall mortality rates of 11.9% for THR and 6.6% for ORIF, without a significant difference [23]. Moreover, the risk of complications such as pneumonia, DVT, and loss of mobility seems to be more dependent on postoperative immobilization than the type of surgical intervention [36]. These and our findings suggest that acute THR can be performed in elderly patients with significant comorbidities without increasing the risk of complications or mortality.

While THR at least does not increasethe risk for adverse events in elderly individuals, the susceptibility to reoperation should be carefully considered given their limited physiological reserves [8]. Previous literature reported an overall revision surgery rate of 14.8% within 10 years after ORIF, compared to 6.2% after acute THR. In addition, 13.5% of patients treated with ORIF required delayed conversion to secondary THR [37]. Other studies have described even higher conversion rates, with approximately 20–25% of elderly patients requiring secondary THR within the first 1–2 years after ORIF [7, 37]. These findings highlight the limitations of ORIF in elderly patients and support considering acute THR as a primary treatment strategy in selected cases to reduce the risk of secondary procedures.

Fracture morphology is one of the most critical determinants for choosing the appropriate treatment strategy to avoid secondary interventions after AF. Acetabular impaction has been identified as the main risk factor for failure of the ORIF procedure [38]. Patients with acetabular dome impaction and comminution had a 23.2% conversion rate to total hip replacement, compared to 9.7% in patients without these injury features [38].

In our cohort, dome impaction was identified in 9 of 20 patients treated with ORIF. Three of these patients (15%) will eventually require secondary THR due to significant signs of osteoarthritis at the end of the observation period, despite achieving anatomical reduction according to Matta’s criteria with a mean MRD of 0.6 mm. In contrast, the THR groups demonstrated even greater fracture complexity, with dome impaction present in 10 of 17 cases and multifragmentary patterns in 12 of 17 cases, yet no reoperations were recorded during follow-up. These findings support the significance of fracture patterns for the optimal choice of the surgical procedure.

Based on our results, THR can also offer better functional short-term results even in patients with highly complex acetabular fracture morphology. In this context, patients treated with SA-THR or CHP showed significantly higher mean HHS at six months compared to those treated with ORIF. Accordingly, previous studies showed that elderly patients treated with acute THR achieve superior functional outcomes compared to ORIF [23]. Weaver et al. reported a mean HHS of 82 in patients treated with THR in comparison to 63 in the ORIF group at a follow-up of approximately six months [39].

Furthermore, patients undergoing THR after previous ORIF demonstrated substantial functional improvement, with the mean HHS increasing from 50 after ORIF to 82 at five-years of follow-up [40]. These findings might be partly explained by the fact that postoperative weight-bearing restrictions in elderly patients are associated with a significantly increased risk of complications and worse functional outcome [4].

In our study, the longer follow-up observed in the ORIF cohort likely reflects the structured rehabilitation pathway required after internal fixation. Weight bearing is advanced in stages, and independent ambulation is permitted only after radiographic confirmation of stability [19]. This contrasts with the arthroplasty protocol, which allows immediate full weight bearing and faster achievement of functional milestones [18]. Although this may introduce a risk of bias in the interpretation of the results, early mobilization was associated with better functional outcomes. Moreover, slower recovery may necessitate extended clinical supervision [41].

While acute THR may offer early mobilization benefits, concerns persist regarding implant longevity and the risk of periprosthetic complications [3]. In the setting of AFs with poor bone quality, a reliable press-fit is often unachievable, requiring supplementary fixation to secure cup stability [10]. Our study revealed promising short-term outcomes, with acceptable acetabular parameters and no cases of dislocation, loosening, or revision within six months. These findings suggest that cup fixation based on SAC anchoring may provide sufficient primary fixation without the need for additional plating in carefully selected cases. A biomechanical study based on this algorithm is currently being developed to further evaluate its validity.

### Study limitations and strengths

This study’s retrospective design inherently carries recognized limitations, including a potential risk of a type II error. The relatively small sample size and single-centre nature restrict the generalizability of the findings. Non-randomized treatment allocation may have introduced selection bias, as the choice between SA-THR, CHP, and ORIF followed a predefined SOP guided by clinical judgment and fracture characteristics.

Patients with insufficient follow-up were excluded from the analysis, which may have introduced attrition bias, as their outcomes could differ from those who completed assessment. Although the standardized six-month evaluation minimized variation in observation time, this duration does not capture long-term complications, implant survival, or fixation integrity.

The available sample provides adequate power to exclude major differences in complication rates, though smaller effects may remain undetected. Therefore, the findings should be interpreted as confirming comparable safety profiles rather than strict equivalence between techniques. The early functional improvements observed after arthroplasty are clinically meaningful but must be viewed within the context of short-term follow-up. Long-term prospective studies with larger cohorts and comparable fracture morphologies are required to validate these results and assess late complications and implant longevity.

Despite these limitations, the study’s strengths lie in its clear decision-making strategies. All procedures were performed by a single experienced surgeon, thereby reducing inter-surgeon variability and enhancing the comparability of the outcomes across treatment groups. Thus, the study provides a comprehensive evaluation of clinical outcomes and standardized functional assessments, including the HHS at multiple postoperative intervals.

## Conclusion

Based on the presented study results, acute THR using a modular revision cup with an iliac peg represents a valuable addition to the treatment spectrum for displaced AFs in elderly patients and offers the advantage of early full weight-bearing with promising functional outcomes. However, the limited 6-month follow-up period restricts these findings to short-term outcomes, and long-term implant stability cannot yet be confirmed. Further biomechanical studies and prospective clinical research are essential to comprehensively validate its long-term safety, durability, and clinical efficacy in larger cohorts.

## Data Availability

The collected data will be stored securely in our institute for 10 years. After 10 years, the data will be deleted. However, all the datasets analyzed or generated during this study will be available from the corresponding author upon reasonable request.

## Abbreviations

AC: Anterior Column
AFs: Acetabular Fractures
AHT: Anterior Hemi-Transverse
AIP: Anterior Intrapelvic Approach
AW: Anterior Wall
BMI: Body Mass Index
CCI: Charlson Comorbidity Index
CHP: Combined Hip Procedure
CHP-Fix: Fixation Stage of Combined Hip Procedure
CHP-Rep: Replacement Stage of Combined Hip Procedure
CoR: Centre of Rotation
CRPIF: Closed Reduction and Percutaneous Internal Fixation
DVT: Deep Vein Thrombosis
HHS: Harris Hip Score
ICD-10: International Classification of Diseases, 10th Revision
ISS: Injury Severity Score
LLD: Limb-Length Discrepancy
LOS: Length of Stay
MRD: Maximal Residual Displacement
ORIF: Open Reduction and Internal Fixation
PACS: Picture Archiving and Communication System
PC: Posterior Column
PHT: Posterior Hemi-Transverse
PW: Posterior Wall
QLP: Quadrilateral Plate
RAA: Roof Arc Angle
RWTH: Rheinisch-Westfälische Technische Hochschule
SA-THR: Stand-Alone Total Hip Replacement
SAC: Supracetabular Corridor
SOP: Standard Operating Procedure
THR: Total Hip Replacement
TTS: Time to Surgery
UTI: Urinary Tract Infection
